# Would We Be Able to Vaildate Diagnostic Criteria and Pave the Way for Discerning Causality in Trigeminal Neuralgia?

**DOI:** 10.5812/kowsar.22287523.3765

**Published:** 2012-01-01

**Authors:** Jeffrey S. Quintana, Hariharan Shankar

**Affiliations:** 1 Department of Anesthesiology, Medical College of Wisconsin, Milwaukee, USA

**Keywords:** Trigeminal Neuralgia, Magnetic Resonance Imaging, Pain

Dear Editor,

Trigeminal neuralgia, a disabling painful disease affects approximately 4-5 per 100,000 individuals per year (1). More recent studies report a much higher incidence of 26 -29 per 100,000 (2, 3). It affects mainly those in the 40-80 years range and when occurring in those younger, secondary causes for trigeminal neuralgia are sought. The American census bureau reported a 15% increase in those 65 years and older between the years 2000-2010 (www.census.gov; last accessed 11/17/11, 7:55pm).

With its predilection for the elderly, the incidence of this disease may be expected to rise.

Validity of any research is based on establishing consistent diagnostic criteria. Presently the diagnosis of trigeminal neuralgia, classical versus symptomatic (when a structural abnormality, like multiple sclerosis or tumors, other than vascular compression is identified), is based on the international classification of headache disorder, second edition, diagnostic criteria for classical and symptomatic trigeminal neuralgia (4). As there are no objective tests, the diagnostic criteria have not yet been validated. Neurophysiological tests may help identify the symptomatic from classical trigeminal neuralgia.

In this issue of Anesthesiology and Pain Medicine, Bangash set out to study the laterality and the common branches of the trigeminal nerve involved in trigeminal neuralgia (1). The diagnosis was established in 100 patients, based on history, clinical examination and therapeutic response to carbamazepine. Subsequent confirmation with imaging and local anesthetic injections, despite limited utility, provided data for analysis. The results, consistent with prior studies with regards to laterality and the commonly affected nerves in trigeminal neuralgia, reiterates the established (5, 6). The author’s novel utilisation of nerve blocks for establishing the involved nerves has certain merits, if localisation can be achieved with certainty. The conclusion of right sided laterality and the mandibular distribution being the commonest for trigeminal neuralgia points to a feature, not yet determined, which may provide a clue to establishing the diagnostic criteria for trigeminal neuralgia. Neurophysiologic testing may reveal trigeminal pathology when not present on magnetic resonance imaging (MRI). Neurophysiologic testing of blink, corneal, and jaw reflexes along with somatosensory evoked potentials, with near nerve stimulation, have shown to be sensitive and reliable indicators of both peripheral and central dysfunction with regards to the trigeminal nerve. Thermal quantitative sensory testing can also be used for detection of trigeminal small fiber dysfunction (7). Improving specificity of diagnostic interventions and the use of right tools i.e. magnetic resonance angiography (MRA) and/or neurophysiological tests, for establishing the diagnostic criteria for trigeminal neuralgia may provide further insight into the reasons behind laterality.
